# Dysfunctions, Molecular Mechanisms, and Therapeutic Strategies of Regulatory T Cells in Rheumatoid Arthritis

**DOI:** 10.3389/fphar.2021.716081

**Published:** 2021-08-26

**Authors:** Xiaoya Li, Huihui Xu, Jing Huang, Dan Luo, Shuang Lv, Xiangchen Lu, Cheng Xiao

**Affiliations:** ^1^ The Institute of Medicinal Plant Development, Chinese Academy of Medical Sciences/Peking Union Medical College, Beijing, China; ^2^ Institute of Clinical Medical Sciences, China-Japan Friendship Hospital, Beijing, China; ^3^ Beijing Key Laboratory of Research of Chinese Medicine on Prevention and Treatment for Major Diseases, Experimental Research Center, China Academy of Chinese Medical Sciences, Beijing, China; ^4^ School of Traditional Chinese Medicine, Beijing University of Chinese Medicine, Beijing, China; ^5^ Department of Ophthalmology, Traditional Chinese Medicine Hospital of Changping District, Beijing, China; ^6^ Department of Emergency, China-Japan Friendship Hospital, Beijing, China

**Keywords:** rheumatoid arthritis, Tregs, molecular mechanisms, dysfunctions, therapeutic strategies

## Abstract

Regulatory T cells (Tregs) represent a distinct subpopulation of CD4^+^ T lymphocytes that promote immune tolerance and maintain immune system homeostasis. The dysfunction of Tregs is tightly associated with rheumatoid arthritis (RA). Although the complex pathogenic processes of RA remain unclear, studies on Tregs in RA have achieved substantial progress not only in fundamental research but also in clinical application. This review discusses the current knowledge of the characterizations, functions, and molecular mechanisms of Tregs in the pathogenesis of RA, and potential therapies for these disorders are also involved.

## Introduction

Rheumatoid arthritis (RA) is a chronic and inflammatory autoimmune disease characterized by persistent synovitis, systemic inflammation, and symmetrical joint involvement. With the development of the disease, osteoporosis also occurs as a complication, and in severe cases, it may develop into a functional disability (Deane and Holers, 2021). Globally, the incidence of RA in adults is 0.5–0.8% according to epidemiological surveys (Lo et al., 2021). Genetic and environmental factors are regarded as the main pathogenesis of RA. In recent years, commensal flora is also recognized as another important pathogenesis of RA (Chiang et al., 2019). Thus far, RA has been mainly treated with disease-modifying antirheumatic drugs (DMARDs), such as methotrexate and leflunomide, supplemented with nonsteroidal anti-inflammatory drugs (NSAIDs) and/or glucocorticoids to alleviate the condition, but it cannot be completely cured. New therapeutic methods, such as biological and cell therapies, are being explored and developed continuously. However, treatments have limited success with this disease.

The immune system maintains a delicate balance between immune activation and immunosuppression through cellular and molecular elements that recognize “cantankerous” antigens to guard the body against attack. Once this balance is disrupted, such as the loss of immunosuppression/self-tolerance, an attack is launched against the autologous tissue, resulting in RA. After regulatory T cells (Tregs) were identified as a specific subpopulation with suppressive capacity maintaining immune homeostasis, numerous studies have related Treg disorders to RA. This review focuses on the molecular mechanism of Tregs in immunosuppression and the roles of these cells in RA.

## History and Basic Understanding of Tregs

Congenitally athymic nude mice have been used as a model of cell-mediated immunological deficiencies for four decades. Based on this model, Pelleitier M. et al. reinforced the hypothesis that spontaneous autoimmunity is partly associated with a T-cell deficiency (Pelleitier and Montplaisir, 1975). However, with continual in-depth research on the subtypes and functions of T cells, researchers have recognized that cellular immunological disorders are correlated with abnormalities in one subtype of T cells but not all kinds of T cells. In 1995, Sakaguchi S. et al. showed that subtype CD4^+^ T cells expressing CD25, which are defined as a functional subset of CD4^+^ non-helper T cells and now recognized as Tregs, maintain immunological self-tolerance, and are associated with various autoimmune diseases (ADs) (Sakaguchi et al., 1995). A work by Powrie F. et al. in 1998 and 2000 showed that interleukin (IL)-10 and cytotoxic T-lymphocyte antigen (CTLA)-4 are essential molecules in the function of Tregs (Asseman and Powrie, 1998; Read et al., 2000). During this time, Seddon B. et al. reported the significance of transforming growth factor (TGF)-β to Tregs (Seddon and Mason, 1999). Immediately after these contributions, Shevach E. M. et al. reported the critical roles of glucocorticoid-induced tumor necrosis factor receptor (GITR) and IL-2 in Tregs in 2002 and 2004 (Mchugh et al., 2002; Thornton et al., 2004). In 2003, forkhead box P3 (Foxp3) was confirmed to play a dominant role in Tregs by multiple researchers, including Sakaguchi S., at the same time (Fontenot et al., 2003; Khattri et al., 2003). Although the recognition of Tregs has been recent, the physiological significance and immunosuppressive function of this subpopulation of T cells have been highly controversial.

At present, the mainstream view is that Tregs are a subgroup of CD4^+^CD25^+^ T cells characterized by the expression of Foxp3. Due to its dominant role in the differentiation, maintenance, development, and functioning of Tregs, Foxp3 has been identified as the master transcription factor of Tregs at early time (Fontenot et al., 2003; Khattri et al., 2003). Tregs comprise approximately 1–3% and 5–10% of CD4^+^ T cell in humans and rodents, respectively. Based on their origin, Tregs can be classified into two subpopulations as follows: thymus-derived Tregs (tTregs), also called natural Tregs (nTregs); and induced Tregs (iTregs) or peripherally derived Tregs (pTregs). The tTregs are produced by the thymus and are functional immunosuppressive T cells due to their stable Foxp3 expression with demethylation of Treg-specific semi-methylated regions at an early stage of differentiation, while iTregs differentiate from naive conventional T cells (Tconvs) in the periphery under certain conditions. Once activated, iTregs show a labile expression of Foxp3. Besides, Tregs can be defined by their function and expression of cell surface markers, and subtypes classified in this manner include activated Tregs (aTregs, CD4^+^CD25^+^CD45RA^−^Foxp3^+^) and resting Tregs (rTregs, CD4^+^CD25^+^CD45RA^+^Foxp3^+^). In addition, a peripheral cell subset that neither expresses Foxp3 nor constitutively expresses CD25, but play the immunosuppressive role through IL-10, is considered to be another Treg cells, named T-regulatory Type-1 (Tr1) cells. Tr1 is not the dominant type in Tregs; thus, we mainly focus on Tregs expressing Foxp3 and CD25 in the review. Although the Foxp3 expression is considered the most common marker and given the non-exclusivity of Foxp3 for Tregs in humans, a negative CD127 expression and even positive GITR expression have been regarded as markers for human Tregs by some researchers in recent years (Ronchetti et al., 2015; Giganti et al., 2021).

## Molecules Related to the Generation, Proliferation, and Functional Maintenance of Tregs

The level of Tregs in the periphery is guaranteed to effectively exert immune tolerance effects. Generation and proliferation of Tregs involve complicated factors, and the main factors are discussed below. The molecules and corresponding functions are represented in Table 1. In addition, there are more mechanisms that regulate the number and function of Tregs, which are described in detail in Qi Jiang et al.’s review (Jiang et al., 2021).

**TABLE 1:** Molecules related to the generation, proliferation, and functional maintenance of Tregs.

Molecules	Function in Tregs	Refs
Foxp3	Development, differentiation, phenotype, and functional maintenance	Chai et al., 2005; Hayatsu et al., 2017
IL-2	Proliferation and functional maintenance	Snow et al., 2003; Setoguchi et al., 2005
TGF-β	Proliferation and functional maintenance	Ghiringhelli et al., 2005; Oettel et al., 2016
GITR	Generation, differentiation, and functional maintenance	Carrier et al., 2012; Mahmud et al., 2014; Petrillo et al., 2015
AhR	Generation, differentiation, and functional maintenance	Gandhi et al., 2010; Ye et al., 2017; Lv et al., 2018; Zamali et al., 2019
Commensal flora		
Bacteria	Generation and functional maintenance	Round et al., 2011; Telesford et al., 2015
Metabolites	Proliferation, differentiation, phenotype, and functional maintenance	Arpaia et al., 2013; Smith et al., 2013; Quintana, 2013; Singh et al., 2014

### Foxp3

Foxp3 is a member of the fork-head/winged-helix transcription factor family, and it encodes a 48-kDa protein named scurfin and shows a selective expression in Tregs. As described above, Foxp3 has been identified as the most common marker and master transcription factor of Tregs, and it plays a vital role in the differentiation, phenotype, and functional maintenance of Tregs. A *Scurfy* gene deficiency leads to autoimmune lymphoproliferative syndrome in mice, while the adoptive transfer of Tregs into newborn mice with a *Scurfy* gene deficiency prevents the occurrence of autoimmune lymphoproliferative syndrome (Fontenot et al., 2003). In humans, both the deletion and mutation (I363V, R397W, and A384T) of the Foxp3 gene induce lymphoproliferative diseases, such as immunodysregulation, polyendocrinopathy, enteropathy, and X-linked syndrome (Gambineri et al., 2003). Moreover, Hayatsu N. et al. illustrated that the A384T mutation alters the interactions between Foxp3 and its specific target genes, including *Batf*, and that sequence-specific perturbations of Foxp3–DNA interactions influence Treg differentiation and accumulation (Hayatsu et al., 2017). In addition, Foxp3 is essential for the development of Tregs from naive CD4^+^CD25^−^ T cells. Retroviral gene transfer of Foxp3 converts naive CD4^+^CD25^−^ T cells into a Treg phenotype similar with that of naturally occurring CD4^+^ Tregs (Chai et al., 2005). Moreover, these cells produce cytokines, including IL-2 and IL-10, upon T-cell receptor (TCR) stimulation, and they express CD25 and CTLA-4, and inhibit the proliferation of effector T cells (Teffs) *via* cell–cell contact but not *via* a cytokine-independent mechanism (Yagi et al., 2004). Altogether, these findings lead to the conclusion that Foxp3 acts as a “master regulator” in Tregs.

### IL-2

IL-2 is crucial for the maintenance and proliferation of Tregs both *in vitro* and *in vivo*. CD4^+^CD25^+^ Tregs constitutively express CD25 (also called IL-2Ra), while Tregs produce low levels of IL-2. Therefore, the development and proliferation of Tregs rely on Teffs. The lack of IL-2, IL-2 receptor-*α* (IL-2R*α*), or IL-2 receptor-β (IL-2Rβ; also called CD122) in mice induces fatal lymphoproliferative diseases accompanied by ADs (Horak et al., 1995). And injecting splenocytes or lymphocytes with IL-2 can prevent these disorders (Malek et al., 2002). A previous study has reported that an anti–IL-2 monoclonal antibody decreases the number of Tregs and inhibits physiological proliferation of these cells (Setoguchi et al., 2005). Signal transducers and activators of transcription 5 (STAT5) is an important molecule in the IL-2 pathway, which activates IL2-signaling by STAT5 phosphorylation. Similar to mice with a defect in the IL-2Rβ expression, STAT5A/5B-deficient mice suffer from a serious multi-organ immune disorder, and the number of Tregs in these mice is significantly decreased (Surbek et al., 2021).

### TGF-β

TGF-β, which binds to heterodimer receptors I and II, phosphorylates *Drosophila* mothers against decapentaplegic protein (Smad) family members, ultimately regulating the transcription of related genes. Upon adequate stimulation, tTregs secrete TGF-β and overexpress TGF-β on the cell surface. There are two types of TGF-β in Tregs, which are as follows: membrane-type TGF-β and secreted-type TGF-β. Secreted-type TGF-β mainly promotes the proliferation of Tregs (Ghiringhelli et al., 2005). TGF-β participates in Foxp3 induction and the proliferation of iTregs by synergizing with IL-2 in the periphery, but it may not be responsible for the Foxp3 expression in tTregs in the thymus. CD4^+^CD25^+^ Tregs from the thymus of dominant-negative TGF-β receptor type II mice retain the ability to inhibit colitis. In contrast, Treg activity in the periphery is suppressed in these mice (Fahlen et al., 2005). In addition, TGF-β mediates the Foxp3 expression by activating the TCR, thus promoting the transformation of Teffs into CD4^+^Foxp3^+^ Tregs (Oettel et al., 2016).

### GITR

GITR (also called TNFSF18 or CD357), a 22-kDa protein belonging to the tumor necrosis factor receptor (TNFR) family, is constitutively expressed on Tregs, and is expressed at higher levels on Tregs (Shimizu et al., 2002). Decades of research have shown that GITR plays a crucial role in the differentiation of tTregs as well as the expansion and suppressive functions of both tTregs and pTregs (Carrier et al., 2012; Mahmud et al., 2014; Petrillo et al., 2015). After engrafting a bone marrow mixture from CD45.2^+^ GITR-deficient mice (Gitr^−/−^) and CD45.1^+^ SJL wild-type congenic mice into RAG2^−/−^ irradiated recipient mice, the mature thymic CD25^+^Foxp3^+^ Tregs generated from the Gitr^−/−^ cells are approximately 70% of those produced from the wild-type cells (Mahmud et al., 2014). In addition, after the administration of GITR ligand (GITRL) antibodies to thymic organ cultures, the development of Tregs and the expression of Treg markers are suppressed (Mahmud et al., 2014). Furthermore, the number of IL-10–producing Tregs is increased in the spleen of GITRL-transgenic mice, which suppresses naive T-cell proliferation in an IL-10–dependent manner (Carrier et al., 2012). More importantly, high expression of GITR on Tregs is beneficial to the outcomes of several ADs, and the correlation between GITR^+^ Tregs and disease severity is more favorable than that between the CD25 expression on Tregs and disease severity based on cumulative studies (Bianchini et al., 2011; Petrillo et al., 2015). In RA, GITR + Tregs expand with an effective treatment, while CD4^+^CD25^+^Foxp3^+^ Tregs cannot achieve a similar expansion (Alunno et al., 2010). Above all, it is not surprising that some researchers urge the scientific community to consider GITR to be a marker of human Tregs. However, it is worth noting that the effect of GITRL/GITR on T cells is context-dependent, specifically depending on the host environment and activation state of the Tregs and Teffs *via* the nuclear factor kappa-B (NF-κB) (Ji et al., 2004; Iwaszkiewicz-Grzes et al., 2021).

### AhR

AhR, the full form for the aryl hydrocarbon receptor, is a cytoplasmic receptor acting in both ligand-dependent and -independent manners, and it can be activated by exogenous and endogenous ligands coming from air pollutants, diet, host metabolism, and the intestinal microbiome. Once activated, the AhR migrates to the cell nucleus and induces transcription of ligand-metabolizing enzymes (cytochrome P1 family) as well as immunoregulatory and growth factors (IL-10, Arginase-1, IL-6, IL-22, and vascular endothelial growth factor) and the negative regulator of the AhR repressor (AhRR) pathway (Denison and Nagy, 2003). In pTregs, AhR is expressed more abundantly, and AhR-expressing Tregs enhance suppressive activity compared with Tregs lacking AhR expression *in vivo* (Ye et al., 2017). *Via* targeting glycolysis and the subsequent nicotinamide adenine dinucleotide (NAD)^+^/Sirtuin (SIRT)1/histone methyltransferase (SUV39H1)/H3K9me3 signaling pathway, the activation of AhR promotes Treg differentiation (Lv et al., 2018). Furthermore, the activation of AhR supports the *de novo* generation of Tregs and promotes the suppressive function in humans (Zamali et al., 2019). While in tTregs, by activating AhR *via* indoleamine 2,3-dioxygenase (IDO) signal, the immunosuppressive function of tTregs is increased (Kumar et al., 2017). In Tr1 cells, the AhR increases the expression of IL-10, granzyme B, and CD33 (a surface molecule contributes to the suppressive function of Tr1 cells). In addition, AhR activation also upregulates the expression of Tr1 autocrine growth factor IL-21 (Apetoh et al., 2010). Interestingly, some research studies found that AhR activation by agonist such as 6-formylindolo [3,2-b] carbazole interfered with the Treg development and increased the severity of autoimmune disease (Quintana et al., 2008). And other research studies got the same result that AhR causes opposite outcomes when activated by 6-formylindolo [3,2-b] carbazole (FICZ) or tetrachlorodibenzo-p-dioxin (TCDD). Thus, AhR regulates Treg differentiation and function in a ligand-specific fashion (Gandhi et al., 2010) through different mechanisms, such as direct functional enhancements, induction of epigenetic modifications of Foxp3, and modulation of dendritic cells (DCs) (Quintana, 2013). Collectively, these findings suggest a role for the AhR in the control of functional Tregs, constituting a unique target for therapeutic immunomodulation.

### Commensal Flora

With the increasing microbiota research studies, the relationship among saliva, respiratory, gut microbiota, their metabolites, and host immunity has been gradually revealed, and it has been found that the alternation of commensal flora is involved in the pathogenesis of RA, chronic colitis, cancer, and diabetes (Lynch and Pedersen, 2016). Among them, the relationship between intestinal flora and disease is the most concerning because intestinal flora not only causes diseases but also cures them. Bacteria and their metabolites enhance immune tolerance in the gut by affecting the differentiation and function of Tregs and partly by the AhR signal pathway.

*Bacteroides fragilis* can produce and export polysaccharide A (PSA), a capsular polysaccharide that is presented to naive CD4^+^ T cells by DCs in the lamina propria (Telesford et al., 2015), and at the same time, PSA through a toll-like receptor (TLR)2 directly helps in the activation of TGF-β–induced Foxp3^+^ Tregs to facilitate the secretion of IL-10 and promote immunologic tolerance (Round et al., 2011; Telesford et al., 2015). Other researchers have demonstrated that *Clostridium Faecalibacterium prausnitzii*, but not related to Clostridia, skew human DCs to prime Tr1-like Tregs by TLR2/6 triggering, c-Jun N-terminal kinase (JNK) signaling, and CD39 ectonucleotidase activity (Alameddine et al., 2019). Besides, *Campylobacterales* can interfere with host immune in different ways. Their flagellins C (FlaC) can activate p38 and decrease the responsiveness of human macrophage-like cells toward the bacterial TLR4 agonist (Faber et al., 2016).

**FIGURE 1:**
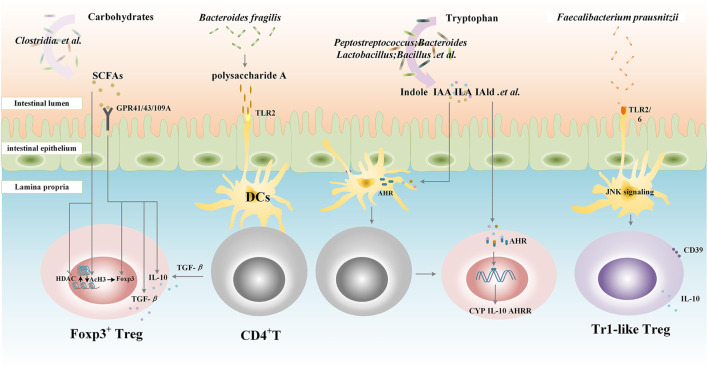
Gut microbiota contributes to the differentiation and function of Tregs. Clostridia metabolize carbohydrates into short-chain fatty acids (SCFAs), which increase Treg numbers, Foxp3 expression, and the suppressive function of Foxp3+Tregs in a histone deacetylases (HDACs)/histone H3 acetylation (AcH3)-dependent manner. SCFAs also induce the differentiation of Tregs and Tr1 cells by increasing the expression of Foxp3 and upregulating the secretion of IL-10 and TGF-β *via* G protein–coupled receptor (GPR) 41, GPCR43 and GPCR109A. *Bacteroides fragilis* exports polysaccharide A (PSA), through a toll-like receptor (TLR) 2; PSA is presented to naive CD4^+^T cells by dendritic cells (DCs) in lamina propria followed by the activation of TGF-β–induced Foxp3^+^Tregs to facilitate the secretion of IL-10 and promote immunologic tolerance. *Bacteroides*, *Bifidobacterium*, *Clostridium*, *Peptostreptococcus*, and *Lactobacillus.* metabolize tryptophan into indole and indole derivatives including tryptamine, indole-3-acetic acid (IAA), indole-3-aldehyde (IAld), and indole lactic acid (ILA), which can activate the aryl hydrocarbon receptor (AHR) translocating to the nucleus and initiating gene transcription (CYP family, IL-10, and AhRR) to enhance the immunosuppressive function of Tregs. Furthermore, the AhR also promotes CD4^+^ cells to differentiate into Tregs by interacting with DCs. *Clostridium Faecalibacterium prausnitzii* skews human DCs to prime Tr1-like Tregs by TLR2/6 triggering, c-Jun N-terminal kinase (JNK) signaling, and CD39 ectonucleotidase activity. SCFAs, short-chain fatty acids; GPR, G protein-coupled receptor; HDACs, histone deacetylases; AcH3, histone H3 acetylation; TLR, toll-like receptor; DCs, dendritic cells; IAA, indole-3-acetic acid; ILA, indole lactic acid; IAld, indole-3-aldehyde; AHR, aryl hydrocarbon receptor; CYP, cytochrome P450; AHRR, AHR repressor; JNK, c-Jun N-terminal kinase; Tr1, T regulatory type-1 cells.

Metabolite studies have found that short-chain fatty acids (SCFAs), including acetic acid, butyric acid, and propionic acid, are the end metabolites of carbohydrates in intestinal flora (such as *Clostridia*) (Furusawa et al., 2013), which can interact with intestinal immune cells and play the role in maintaining immune tolerance to the intestinal flora. As the inhibitors of histone deacetylases (HDACs) and enhancers of histone H3 acetylation (AcH3), SCFAs increase Treg numbers, Foxp3 expression, and the suppressive function of Foxp3^+^Tregs in an HDAC-dependent manner (Arpaia et al., 2013). In addition, as ligands for G protein–coupled receptors (GPCRs), SCFAs induce the differentiation of Tregs and Tr1 cells by increasing the expression of Foxp3 and upregulating the secretion of IL-10 and TGF-β *via* GPCR41, GPCR43, and GPCR109 (Smith et al., 2013; Singh et al., 2014). Intestinal flora can also regulate the function of immune cells through other metabolites such as tryptophan derivatives. Tryptophan, an essential amino acid provided by dietary protein, is metabolized by several bacteria such as *Bacteroides*, *Bifidobacterium*, *Clostridium*, *Peptostreptococcus*, and *Lactobacillus.* Among metabolites from tryptophan, indole, and its derivatives, tryptamine, indole-3-acetic acid (IAA), indole-3-aldehyde (IAld), 3-methyl-indole (skatole), indole-acrylic acid (IA), and indole lactic acid (ILA) are AhR ligands (Roager and Licht, 2018). Once activated, the AhR translocates to the nucleus and initiates gene transcription (cytochrome P450 (CYP) family, IL-10, and AhRR) to enhance the immunosuppressive function of Tregs (Denison and Nagy, 2003). Furthermore, the AhR also promotes CD4^+^ cells to differentiate into Tregs by interacting with DCs (Quintana, 2013) (Figure 1).

The immune regulation of bacteria in hosts is currently a hot research topic. More studies on the immune regulatory mechanism of pathogenic bacteria and probiotics are still needed. More importantly, whether basic research can be turned into clinical practice to benefit patients will be the focus in the early future.

## Molecular Mechanism of the Immunosuppressive Function of Tregs

The dominant function of Tregs is the control of all aspects of the immune response. Antigen presenting cells (APCs), such as DCs, macrophages, and B lymphocytes, are the primary targets of Tregs, and responder T cells, such as CD4^+^T cells Th cells, CD8^+^ T cells, and natural killer (NK) T cells, are efficient targets of Tregs. In addition, mastocytes, osteoblasts, and osteoclasts (OCs) are targets of Tregs. The mechanisms regulating these cell types are broadly classified as targeting T cells (the secretion of suppressive cytokines, disruption of metabolic processes, and induction of apoptosis) and targeting APCs (decreased co-stimulation or decreased antigen presentation).

### Expression of Suppressive Cytokines

Tregs secrete suppressive cytokines, including IL-10, TGF-β, and IL-35. As previously described, TGF-β plays a critical role in the induction of Tregs both *in vivo* and *in vitro*, while the contribution of TGF-β to the suppressive capacity of Tregs remains controversial. A mount of studies using humans, mice, or cultured cells have failed to illustrate that TGF-β, either in the membrane-bound or soluble form, is essential for Treg-mediated suppression (Piccirillo et al., 2002; Kullberg et al., 2005; Oberle et al., 2007). In contrast, mice without T-cell–specific TGF-β production or processing develop an autoimmune syndrome (Li et al., 2007). In addition, Nakamura K. et al. suggested that membrane-type TGF-β–mediated suppression may inhibit Teffs by binding to the TGF-β receptor on the Teff surface, and Hui X. et al. observed that triptolide restrains OCs differentiation and bone resorption *in vitro* through IL-10 and TGF-β1 production by Tregs (Xu et al., 2016). Thus, we proposed that the role of TGF-β in Treg-mediated suppression may depend on the type of Teffs and the site of the immune response in a cell contact–dependent manner (Andersson et al., 2008). IL-10 production by Tregs is essential for restraining immune responses, such as those in the bone and those involved in mucosal homeostasis (Li et al., 2016a). For instance, Asseman C. et al. illustrated that colitogenic Th1 cells are suppressed by CD4^+^CD25^+^ Tregs in an IL-10–dependent manner in mice (Asseman and Powire, 1998). Furthermore, an anti–CTLA-4 mAb induces high levels of IL-10 secretion by Tregs and high expression of inducible co-stimulator (ICOS) on Tregs, and this treatment inhibits Th1 memory responses and represses experimental intestinal inflammation in a mouse model of colitis (Coquerelle et al., 2009). In APCs, IL-10 suppresses the production of pro-inflammatory cytokines, chemokines, and chemokine receptors, as well as the expression of major histocompatibility complex (MHC) class II and CD80/CD86 co-stimulatory molecules (Akdis and Akdis, 2014). Another cytokine, IL-35, contributes to the function of Tregs by directly affecting responder T cells such as T helper cells 1 (Th1) and 17(Th17), which likely affects DCs (Song and Ma, 2016).

### Induction of Apoptosis

Granzyme A, granzyme B, and perforin are three of the key components of Treg-induced suppression mediated through the induction of apoptosis in a cell–cell contact manner (Gondek et al., 2005). Tregs mediate cytotoxicity-induced cell death through the CD95 death receptor and caspase 3 in multiple cell types, including CD4^+^ T cells, CD8^+^ T cells, CD14^+^ monocytes, NK cells, and DCs, in humans (Grossman et al., 2004; Ataera et al., 2013). In addition, galectin-1, which is secreted by Tregs, binds to glycoproteins (CD45, CD43, and CD7), resulting in cell cycle arrest and apoptosis in responder cells (Garin et al., 2007). The other mechanism is related to IL-2, and this mechanism is controversial. Some groups have demonstrated that the consumption of IL-2 by Tregs induces apoptosis in effector cells in mice (Pandiyan et al., 2007). However, Oberle did not observe the induction of apoptosis in Tconvs by Tregs in humans (Oberle et al., 2007). More recent studies have shown that Tregs suppress CD8^+^ T-cell responses *via* IL-2 depletion and that IL-2 plays an indispensable role in mice (Vercoulen et al., 2009). Based on the findings above, it is thought that the consumption of IL-2 by Tregs diminishes the IL-2 pool that is indispensable for Teffs, resulting in restricted expansion and even leading to apoptosis. Additionally, other studies have reported that IL-2 contributes to the development of the suppressive function of Tregs through calcium signaling to T and NK cells in patients (Whitehouse et al., 2017). Thus, we speculate that IL-2 activates multiple signaling pathways to suppress functions and/or induce apoptosis in target cells and that differences are likely due to cell types.

### Disruption of Metabolic Processes

Tregs disrupt metabolic processes in target cells. One mechanism is mediated through the CD39/CD73 expression, and it results in the metabolism of adenosine triphosphate (ATP) to adenosine 5′-monophosphate (AMP) and the production of adenosine, which binds to the adenosine A2A receptor, followed by an increase in the intracellular cyclic adenosine 3, 5′-monophosphate (cAMP) level in target cells (Tconvs and DCs) (Borsellino et al., 2007; Ohta and Sitkovsky, 2014). Subsequently, the protein kinase A (PKA) pathway and the noncanonical cAMP pathway are activated, resulting in immunosuppression.

### Decreased Costimulation and Antigen Presentation by APCs

Tregs decrease costimulation and antigen presentation by regulating the cell contact–dependent modulation of APCs. CTLA-4, which is located on the surface and in the submembrane vesicles of Tregs, is also detected in Golgi vesicles of Tconvs at rest and released after the stimulation of the TCR (Tai et al., 2012). The molecular mechanism of Treg-mediated suppression *via* CTLA-4 is clear in relative terms. For APCs, particularly DCs, CTLA-4 competes with the CD28 co-stimulatory molecule for binding to the CD80/CD86 molecules to induce cell cycle arrest (Chikuma, 2017). Furthermore, CTLA-4 downregulates the CD80/CD86 expression on APCs through trans-endocytosis to directly suppress Tconvs *in vitro* and *in vivo* (Qureshi et al., 2011). In addition, Tregs facilitate the DC expression of IDO *via* CTLA-4, which converts tryptophan to kynurenine in Teffs, and this change also leads directly to cell cycle arrest and reduced glutathione synthesis, resulting in a redox environment detrimental to Tconv proliferation (Yan et al., 2010). Furthermore, an increased IDO expression promotes iTreg generation (Curran et al., 2014). A recent report has illustrated that Tregs suppress autophagy in DCs *via* CTLA-4–mediated activation of the PI3K/Akt/mTOR axis and FoxO1 nuclear exclusion, subsequently leading to decreased transcription of the autophagy component, microtubule-associated protein 1 light chain 3*β* (Alissafi et al., 2017). Lymphocyte activation gene (LAG)-3 (CD223), a CD4 homolog that binds to MHC class II on immature DCs, induces immunoreceptor tyrosine-based activation motifs (ITAM)-mediated inhibition to suppress the development and antigen presentation capacity of DCs (Liang et al., 2008).

## Tregs in RA

### Mechanisms of Abnormal Treg Level

#### Level of Tregs in RA

The proportion of Tregs in the peripheral blood of patients with RA is controversial. Some studies have shown that RA patients have lower or higher frequencies of CD4^+^CD25^+^Foxp3^+^Tregs or CD4^+^CD25^+^CD127^−^Tregs in the peripheral blood than healthy subjects (van Roon et al., 2010; Vitales-Noyola et al., 2018). Other researchers have found that the level of peripheral CD4^+^CD25^+^Foxp3^+^Tregs in RA patients is almost the same as that of healthy people (Tang et al., 2011). Based on the existing cognitive status of RA immunity, the number of Tregs should be decreased in RA. As shown in Supplementary Table 1, a review of the literature from the past 10 years indicates that most studies do report that the level of peripheral Tregs is decreased in RA patients.

Based on our review, there are several reasons leading to the different Treg results. 1) Different markers lead to different ratios. Although CD4^+^CD25^+^Foxp3^+^ cells are thought to be the underlying Tregs, markers of Tregs remain controversial, and researchers may use different markers for different purposes. For example, Yang M et al. not only studied the proportion of CD4^+^CD25^+^CD127^low/−^foxp3^+^Tregs but also observed CD4^+^CD25^+^CD127^low/−^Foxp3^+^Helios^+^Tregs, CD4^+^CD25^+^CD127^low/−^Foxp3^+^CD226^+^ Tregs, and CD4^+^CD25^+^CD127^low/−^Foxp3^+^ TIGIT^+^ Tregs in the same study and obtained different results (Yang et al., 2019). 2) Similarly, different Treg phenotypes have different proportions in RA. Altered Tregs may be just one of the phenotypes of Tregs, while the other phenotypes remain unchanged. For example, a previous study has shown that the aTregs (CD4^+^CD25^+++^CD45RA^−^) and not the rTregs (CD4^+^CD25^++^CD45RA^+^) contribute to the decrement of total Tregs in the peripheral blood of RA (Kim et al., 2012). 3) The percentage of Tregs may be related to the disease state. Compared to the remission stage, the active stage of the disease is mainly characterized by intense inflammatory activity and causes more abnormal immune status. Wu et al. studied this point and found that the proportion and function of peripheral blood Tregs in remission RA are almost the same as in the normal control, but that the proportion and function represented by IL-10 Tregs are decreased in active RA (Wu et al., 2016). 4) Furthermore, the different results may be related to the duration. Barbera et al. reported that unchanged Tregs are present in early RA (Barbera et al., 2016). Another study has found that patients with a mean (range) RA duration of 15.2 (2–79) months now show a difference in peripheral blood Tregs compared to the control but that patients with a mean (range) disease duration of 123.7 (13–300) months have significantly lower peripheral blood Tregs than the control group. 5) In addition, the age of RA patients included in the study may contribute to the difference in the Treg status. Pawłowska J. et al. researched the relationship of age of RA onset with status of T cells and found that older age of RA onset is associated with a higher level and the activation status of peripheral blood CD4^+^ T cells, including Tregs and disease activity (Pawlowska et al., 2011).

In the study of Tregs, surface molecular markers are a common method for the classification of Tregs, but they may be deviant due to the influence of reagents and operating procedures. The search for new markers, such as specific miRNA expression, may resolve the confusion of markers to a certain extent. Furthermore, the age, disease course, and disease status of patients should be fully evaluated when RA patients are included.

#### Molecular Mechanisms of Abnormal Treg Level

RA patients maintain a continuous inflammatory state, and inflammatory factors such as IL-6, TNF*-*α, and IL-1β are significantly increased. The persistent inflammatory state inhibits Treg formation and Foxp3 expression. Studies have shown that the levels of Tregs and IL-6 in peripheral blood mononuclear cell (PMBC) are negatively correlated (Khoshmirsafa et al., 2018). In addition, the activated NF-κB pathway by inflammatory environment can drive miR-34a–impairing Treg/Th17 balance *via* targeting Foxp3 (Xie et al., 2019).

The level of exosomes in peripheral blood is changed in RA patients, and altered exosomes selectively affect Tregs. Wang et al. found that several miRNAs in the exosomes from RA peripheral blood were more abundant than those from healthy control donors. In upregulated miRNAs in RA patients, miR-17 inhibits induction of Tregs and reduces the number of Tregs by inhibiting the expression of TGF receptor II (TGFBR II) (Wang et al., 2018).

In addition, the abnormal number of Tregs is also caused by abnormal intestinal flora. As previously described, much bacteria produce metabolites that activate AhR, which plays an important role in the *de novo* production of Tregs. AhR expression is low on Tregs in RA, and Treg level can be significantly increased by activating the AhR pathway. A number of studies have shown that the abundance of bacteria that produce AhR ligands decreases in RA, and the activity of the AhR pathway in Tregs also decreases. By improving the colony structure of intestinal flora, the abundance of bacteria that metabolizes AhR ligands can be called back, followed by the increasing activity of the AhR signaling pathway, and upregulating the number of Tregs (Li X. et al., 2020). Besides, other cells involved in Treg generation, such as DCs and B cells, are abnormal in RA, which results in impaired Treg formation justifiably (Moore et al., 2010).

### Mechanisms of Impaired Function of Tregs

#### Unique Function of Tregs in RA

Interaction with Th17. The balance between Tregs and Th17 is the key point in the maintenance of immune homeostasis. Similarities to that of other ADs, such as systemic lupus erythematosus and autoimmune hepatitis, the pathogenesis of RA has been observed to involve an imbalance between Tregs and Th17 (Jin et al., 2021). The inflammatory cytokine environment is a key driver that could globally push Tregs/Th17 toward imbalance because the combination of TGF-β and IL-6 allows T-cell differentiation toward the Th17 phenotype, whereas if TGF-β is present alone, T cells will differentiate or revert into iTregs *in vitro* (Bettelli et al., 2006). Further research has shown that bacterial metabolites, dietary ligands of AhR, have immunomodulatory effects of differentiation of Tregs and Th17 in multiple ADs including RA (Yuan et al., 2016). In addition, our group also found that by activating the AhR signal, the human umbilical mesenchymal stem cells (HUMSCs) display therapeutic potential in RA by regulating immune imbalance dominated by Tregs and Th17 (Li M. et al., 2020). Further study found that AhR activation also decreases Th17 cytokines and increases IL-10 expression (Kim et al., 2019).

Involved in bone immunity. In addition to the function of immunosuppression, Tregs also show other functions specific to the disease characteristics of RA. Tregs play an important role in alleviating cartilage damage and bone destruction in RA. As a characteristic secondary disease, osteoporosis is common in RA patients. Cartilage damage and bone destruction in RA are partly caused by the activation of OCs. Previous studies have implied that Tregs suppress OC differentiation and bone resorption by regulating IL-10, TGF-β1, and IL-4 (Xu et al., 2016). Furthermore, a previous report had shown that in addition to IL-10, TGF-β1, and IL-4, CD80/86-deficient OCs cannot be inhibited by CTLA-4 or Tregs, suggesting that CTLA-4 and CD80/86 effectively inhibit OCs by inducing IDO/tryptophan (Bozec et al., 2014). Among them, the metabolites from the IDO/tryptophan pathway are the main endogenous ligands in regulating AhR (Kumar et al., 2017). Based on this, it is speculated that AhR is the signaling pathway in Treg-regulated bone immunity. Based on the OC-Treg co-culture system *in vitro*, our previous research found that Tregs inhibit OC differentiation and bone resorption in a quantity-dependent manner, and triptolide enhanced the bone immunity function of Tregs *via* producing the IL-10 and TGF-β1 (Xu et al., 2016). HUMSCs alleviate the destruction of bones *in vivo* by shifting Th1 cells toward the Th2 phenotype, inducing Tregs and increasing the expression of IL-10 and TGF-β1 in a collagen-induced arthritis (CIA) model (Li X. et al., 2020).

#### Symptoms of Functional Impairment of Tregs

Obviously, the function of Tregs plays an important role in the prognosis and deterioration of RA. Among the studies that have researched the immunosuppressive function of Tregs, however, most studies found that the function of Tregs is reduced as indicated in Supplementary Table 1.

The function of Tregs is impaired in many ways. Numerous studies have shown that Tregs derived from RA patients have a decreased ability to inhibit the proliferation and inflammatory factor secretion from Teffs (van Roon et al., 2010; Cribbs et al., 2015). Another study has found significant reductions in Treg secretion of IL-10 and TGF-β1 in RA patients (Hashemi et al., 2018). Furthermore, studies have shown that Treg apoptosis in RA patients is increased and that Tregs are uncontrolled due to B-cell apoptosis mediated by Fas in RA (Li et al., 2014; Rapetti et al., 2015). In addition, the reduced function of Tregs may also be reflected in the reduced ability to interfere with the metabolic processes of target cells. As previously explained, one way in which Tregs play an inhibitory role is through the disruption of metabolic pathways, and CD35/CD27 is an important molecule on the surface of Tregs. One study has found that the expression of CD39 and CD73 is decreased in Tregs from RA patients (Zhang et al., 2019). CTLA-4 expression is also downregulated in Tregs from RA patients (Aldridge et al., 2021), which results in decreased co-stimulation and antigen presentation, indicating that the CTLA‐4–mediated cell cycle arrest of APCs by Tregs is also weakened.

#### Molecular Mechanisms of Tregs Impaired in RA

Further studies have revealed the mechanisms that how Tregs are impaired. Similar with the abnormal levels of Tregs in RA, long‐term inflammatory environment interferes with the immunosuppressive function of Tregs. Especially, high concentrations of TNF‐α in RA patients appear to interfere with the suppressive function of Tregs (Farrugia and Baron, 2016). This evidence supports the excellent efficacy of TNF‐α inhibitors for RA. Besides, in an inflammatory environment, Teffs are resistant to Tregs (Walter et al., 2016), which also exacerbates the immune imbalance.

An abnormal Foxp3 gene expression is also an important factor for abnormal Treg function. Hashemi et al. found that polymorphism of the Foxp3 gene affects the frequency and function of Tregs in RA patients, and RA patients with the AA genotype have lower frequencies of Tregs, and levels of TGF-β and IL-10 than patients with CC and CA genotypes (Hashemi et al., 2018). Kennedy A. et al. identified a novel differentially methylated region (DMR) in upstream of the Foxp3 promoter, which exhibits dysregulated methylation in RA Tregs. Cribbs AP. et al. further proved that demethylation of the Foxp3 upstream enhancer restores Treg function in RA patients (Cribbs et al., 2015). In addition, phosphorylation of Foxp3 controls the function of Tregs, which can be inhibited by TNF-α in RA (Salomon, 2021). Besides, Su Q. et al. found the development of Tregs in RA was related to impaired Tip60-mediated Foxp3 acetylation (Su et al., 2019).

Besides, an inadequate expression of cell surface molecules and failure to produce the soluble factors induce dysfunction of Tregs. The decreased expression of CTLA-4 in Tregs of RA is compromised by CTLA-4 promoter methylation, resulting in a failure to activate the indoleamine 2,3-dioxygenase pathway (Cribbs et al., 2014). Therefore, CTLA-4-Ig therapy enhances the function of Tregs in patients with RA.

Moreover, phenotypic change in Tregs is common in RA patients. A previous study has reported that a higher frequency of IL-17–producing Tregs is present in the peripheral blood of RA patients than in healthy subjects (Wang et al., 2015). In addition, an increased number of senescent Tregs in RA patients also contributes to the decreased function of Tregs (Fessler et al., 2017).

In the mechanism study of Tregs in RA, most researchers focus on how Tregs work, while few scholars focus on what influences Tregs’ work. We think understanding why impaired function of Tregs in RA is better for restoring RA’s immune homeostasis.

### Treg-Based Strategies for RA

In consideration of the powerful immunosuppressive function of Tregs, Treg-based therapies become one of the major directions in RA treatment, and several therapeutic strategies have been explored. Although population heterogeneity and instability impact the curative effects of Treg-based strategies, these approaches have had a significant positive impact on the recovery of RA patients in recent years.

#### Conventional Drug Therapy

DMARD. To the best of our knowledge, there are no specific Treg-targeted chemical drugs or herbal medicines being used in the clinic or basic research, but many drugs show a beneficial effect on Treg levels and function. Methotrexate, a traditional DMARD, is one of the gold standards of therapy for RA that restores defective Treg function through demethylation of the Foxp3 locus, leading to subsequent facilitates in Foxp3 and CTLA-4 expression (Cribbs et al., 2015). Other widely used DMARDs, both leflunomide and sulfasalazine, inhibit the antiproliferative function of Tregs on co-cultured Teffs, and they reduce the Treg expression of Foxp3 mRNA in PBMCs from healthy adults (Oh et al., 2013), and leflunomide can increase Treg cells but reduce Th17 in PBMC *via* activating AhR (Baban et al., 2012). In addition, iguratimod, an approved drug in the treatment of RA, downregulates the Th1/Th17-type response and upregulates Tregs. At the same time, associated cytokines and transcription factors are also changed in a similar trend (Xu et al., 2015).

Biologicals. As more and more mechanisms are studied, biological therapies targeting to Treg number, function, and differentiation are being utilized to treat RA. TNF-α impairs the differentiation and function of TGF-β–induced Tregs in RA through the Akt and Smad3 signaling pathways, and the specific TNF-α inhibitor, infliximab, has been reported to increase the number of peripheral Tregs, especially through the generation of TGF-β–induced Tregs in RA patients (Salomon, 2021). Furthermore, TGF-β–induced Tregs are completely resistant to Th17 by IL-6 conversion and maintained the immunosuppressive effect, while tTregs lost most of their cellular function, indicating that the dysregulation of IL-6 plays an important role in Treg function (Niu et al., 2018). Tocilizumab, an anti–IL-6R antibody, has become a strong and effective therapy for RA by regulating the proportion of Tregs in the PBMC population (Li et al., 2016b). Moreover, numerous studies have shown that a low dose IL-2 promotes balance among the Th1, Th17, and Treg distributions in patients with progressive RA (Kosmaczewska et al., 2015). Interestingly, IL-2 not only promotes naive CD4^+^ T-cell conversion into Tregs but also drives the differentiation to Teffs. The dominant cell type impacted depends on the dose of IL-2 because Tregs require 10–20 times less IL-2 than Teffs at the p-STAT level and 100-fold less at genetic transcription levels. Therefore, whether IL-2 has a positive or negative impact on RA depends on the IL-2 concentration (Klatzmann and Abbas, 2015). Additionally, CTLA-4-Ig therapy enhances the function of Tregs in patients with RA (Alvarez-Quiroga et al., 2011).

Although biologics have pitfalls, they are promising. The development of biological agents with multiple targets, low side effects, and high reactivity is imminent.

#### Traditional Chinese Medicines

Although conventional drugs have been relatively systematic and effective, these drugs are reported to have varying degrees of side effects, such as gastrointestinal disorders, immunodeficiency, infection, and humoral disturbances. Exploring new therapeutic agents with low toxicity and high efficacy is strongly needed in the treatment of RA. Complementary therapies based on TCM can be recommended as complementary and alternative.

Formula. The traditional Chinese medicine formula focuses on individual treatment, which is based on the guidance of traditional Chinese medicine theory and compatible with different Chinese herbs. The traditional Chinese medicine formula plays a multi-target therapeutic role by regulating the system state of the human body, with definite curative effect and low side effect. Traditional Chinese medicine formula plays an indispensable role in the treatment of RA in China. Yi Shen Juan Bi Pill, a traditional Chinese medicine formula widely used in clinics, has powerful regulatory effects in CIA rats *via* regulating Tregs (Zhao et al., 2018; Peng et al., 2019). Another decoction named Qianghuo Erhuang can significantly decrease the disease activity score in 28 joints (DAS28), erythrocyte sedimentation rate (ESR), and C-reactive protein (CRP) in RA and upregulate the percentage of Tregs in adjuvant-induced arthritis in rats (Qian et al., 2017). Besides, Er Miao San attenuates rat’s arthritis by regulating Th17/Tregs (Dai et al., 2020).

Extract. Other drugs, especially Chinese herbal extracts, also have the potential to regulate Tregs in experimental studies. Among these, total glucosides of paeony, from *Radix Paeoniae* Alba, dynamically regulate gut microbiota, suggesting that the regulatory role of Tregs is related to the change of intestinal flora. *Tripterygium wilfordii* glycosides, extracted from *Tripterygium wilfordii* Hook.f*.*, demonstrate great improvement in RA disease activity and immunosuppressive *via* Tregs (Luo et al., 2019). In addition, ethanolic extract of the *Saussurea lappa* (costus) root and water extract of the *Saussurea involucrata (Kar. et Kir.) Sch.-Bip.* have potential anti-arthritic activity and improves the immune responses of adjuvant-induced monoarthritis in rats (Han et al., 2016; Tag et al., 2016).

Natural compound from TCM. One study has reported that oxymatrine significantly reduces the production of TNF-α and IL-17A but upregulates Foxp3 expression in CIA rats (Ma et al., 2017). In addition, periplocoside A increases the proportion of Tregs among helper T cells and inhibits the differentiation and reactivity of h in CIA mice (Yang et al., 2017). Grape seed pro-anthocyanidin has proven potent anti-arthritic effects on CIA by inducing Th17/Treg rebalance (Ahmad et al., 2013). Dioscin, extracted from *Dioscorea nipponica* Makino, affects the differentiation of Tregs, secretion of related factors, and the expression of STAT3 and STAT5 in CIA mice (Xing et al., 2019). In the gut lymphoid tissues of sinomenine-treated rats, the frequency of Tregs is facilitated, and the frequency of Th17 is decreased (Wang et al., 2013). Based on this study, it is speculated that the regulatory effect of sinomenine on Tregs may be related to intestinal flora. The percentage of AhR^+^ cells in CD4^+^CD25^+^T cells has been proven to be significantly lower in RA patients than in controls (Cheng et al., 2017). As a ligand of the AhR, tetrandrine and flavonoid naringenin mediate Tregs through the AhR pathway (Wang W. et al., 2012; Yuan et al., 2016).

The curative effect of TCM is great, but due to the complex composition, unclear mechanism, a small part of traditional Chinese medicine with side effects, and other reasons, TCM’s recognition is limited. In addition, the use of traditional Chinese medicine needs the guidance of the basic theory of traditional Chinese medicine, which also limits the wide application of them.

#### Bacteria-Based Therapy

Specific alterations are observed in the gut and oral microbiomes in RA individuals and CIA models. Furthermore, studies have been done to demonstrate the influence of flora on RA by administering pathogenic bacteria *Porhyromonas gingivalis* that can aggravate rheumatoid arthritis (Sato et al., 2017). Consequently, it is hoped that changes in intestinal flora in a certain direction might be effective in RA treatment. Currently, there are roughly two therapeutic methods for affecting gut microbiomes.

One is to observe the changes of gut microbiomes in RA or animal models through agent intervention, such as total glucosides of paeony and HUMSCs. This method cannot completely prove that the efficacy is caused by the change of flora because it is possible that the host immune state is change first, followed by the change of gut microbiomes affected under the host intestinal mucosal immunity.

Another is microbiota transplantation. There are also two types of microflora transplantation, which are as follows: one is oral prebiotics and probiotics or a combination of the two synbiotics, and the other one is fecal microbiota transplantation (FMT). Supplementation of synbiotics had beneficial effects among patients with RA. And supplementation of probiotics, such as *Lactobacillus casei*, decreases disease activity and increases the release of IL-10 of RA as reported in a randomized double-blind clinical trial (Alipour et al., 2014). In addition, *Lactobacillus salivarius* and *Lactobacillus plantarum* isolated from RA patients increase Treg frequency, and decrease arthritis scores, synovial infiltration, and bone erosion in CIA mice (Liu et al., 2016). In addition, synbiotics, PSA, derived from the human commensal *Bacteroides fragilis* can stimulate immunologic development, which can increase suppressive function and promote differentiation of Tregs (Telesford et al., 2015). Furthermore, heat-killed *Lactobacillus reuteri* alleviate the severity and the prevalence of CIA by increasing the frequency of Tregs and CD4^+^IL-10^+^ cells, which means the probiotic supplementation can not only treat the RA but also prevent the RA (Yokota et al., 2018). As for FMT, the rise of FMT is due to its excellent efficacy in the treatment of *Clostridium difficile* infection, and its subsequent efficacy is shown in other inflammatory diseases such as inflammatory bowel disease and pseudomembranous enteritis with unknown pathogen infection. Up to now, there is no report on the efficacy of FMT in RA. The closest study is a 6-month, double-blind, randomized, placebo-controlled trial in patients with psoriatic arthritis (Kragsnaes et al., 2018). Despite the risks of FMT, its immunomodulatory effects in RA should be fully exploited.

Studies on microflora focus on intestinal microflora, but oral microflora and ectopic microflora should also be investigated. In addition, there is no research on RA treatment by FMT, which may be a new direction for the development of RA therapy.

#### Cell Transfer Therapy

Given its fewer side effects and potential efficacy, cell transfer therapy is being considered the epoch of a new generation of therapy for treating a multitude of diseases, including RA, distinct from the era of treatment with pharmacological agents. Although the curative effect of Treg transfer is limited under some conditions, several studies have revealed that Treg transfer prevents the progression of RA (Niu et al., 2018). After supplementation with chemical reagents to modify Treg signaling pathways or biological agents for genetic modification, the purity, yield, specificity, and safety of Treg treatments are enhanced (Milward et al., 2017). Except for a few clinical studies, the Treg transfer therapy is mostly in the stage of animal studies. It is worth mentioning that this therapy has been successful in CIA. After transferring Tregs into immunocompetent CIA mice, disease progression is slowed, and Tregs are found in the inflamed synovium soon after transfer (Morgan et al., 2005). Beyond transferred Tregs, stem cell transplantation shows an immunoregulatory capability during the progression of RAs based on the Treg function. Specifically, the use of HUMSCs has shown promising results relating to a significant increase in Treg percentage in clinical trials even in severe and resistant RA patients (Ghoryani et al., 2019). Our previous study has also suggested that HUMSCs alleviate CIA partly by regulating Tregs and Th17 (Li M. et al., 2020).

Cell transfer therapy appears to be safe and well tolerated, but the challenge is to obtain stable function and a substantial number of Tregs that are of controlled quality.

#### Target-Based Therapy

The most successful example of target-based therapy is tofacitinib (CP-690,550), which is a blockbuster drug affected in patients with moderately to severely active RA who have had an inadequate response or intolerance to methotrexate. Tofacitinib is a selective Janus kinase (JAK) inhibitor that preferentially inhibits JAK1 and JAK3. Cytokine binding to receptor of JAKs activates JAKs, which phosphorylates signal transducers and activators of transcription (STATs). Then, JAKs and STATs dimerize and translocate to nucleus where they regulate gene transcription (Ptacek et al., 2021). By blocking interferon (IFN) and IL-6, tofacitinib can decrease synovial immune and inflammatory responses (Boyle et al., 2015). At a clinically relevant dose, tofacitinib effectively preserves the suppressive activity of CD4^+^CD25^bright^ Tregs but inhibits Teff functions by suppressing IL-2–induced P-STAT activity (Sewgobind et al., 2010).

Furthermore, as a key target connecting host immunity and intestinal flora as described above, AhR might be an effective target for regulating both immune status and intestinal flora. In another autoimmune disease, AhR agonists have been effective in plaque psoriasis on a Phase 2, randomized dose-finding study (Robbins et al., 2019). The result suggests the potential of AhR agonists in the treatment of RA.

## Summary and Future Perspectives

Although there is a considerable amount of knowledge about Tregs, continuous and repeated courses of RA with high disability rates are still occurring. Most studies on Tregs mainly focus on the number/proportion and associated cytokines, but the immunosuppressive mechanism of Tregs is much more complicated and study worthy. A previous study has made it clear that the pathogenesis of RA and prognosis are related to genetic and environmental factors. The curative effect of drugs on the individual differences in the corresponding relationship with a genotype is helpful to guide the choice of drugs and accurate medicinal development. However, few studies have focused on drug research based on genotype. In addition, several researchers are studying the effects of environmental pollutants, namely, polycyclic aromatic hydrocarbons, and their receptor AhR on RA and Tregs. Fortunately, tapinarof may have potential to treat RA (Bissonnette et al., 2021). Although single-target therapy for single molecules and single proteins is effective, the efficacy is limited. Individualized and system-based treatments should be a greater focus. A rational application of TCM, precise treatment based on genotype, and bacteria-based therapy may be the future of RA treatment.
