# Supplementary material for: Dysfunctions, Molecular Mechanisms, and Therapeutic Strategies of Regulatory T Cells in Rheumatoid Arthritis
Source: Front Pharmacol. 2021 Aug 26;12:716081. doi: 10.3389/fphar.2021.716081 (PMC8428974; doi:10.3389/fphar.2021.716081)
Supplement: Supplementary file 1 [file Table1.docx]

Supplementary Material

Dysfunctions, Molecular Mechanisms, and Therapeutic Strategies of Regulatory T Cells in Rheumatoid Arthritis

Xiaoya Li^1,2#^, Huihui Xu^3#^, Jing Huang^4^, Dan Luo^5^, Shuang Lv^2,4^, Xiangchen Lu^2,4^, Cheng Xiao^2,6*^

^1^ The Institute of Medicinal Plant Development, Chinese Academy of Medical Sciences/Peking Union Medical College, Beijing, China

^2^ Institute of Clinical Medical Sciences, China-Japan Friendship Hospital, Beijing, China

^3^ Beijing Key Laboratory of Research of Chinese Medicine on Prevention and Treatment for Major Diseases, Experimental Research Center, China Academy of Chinese Medical Sciences, Beijing, China

^4^ School of Traditional Chinese medicine, Beijing University of Chinese Medicine, Beijing, China

^5^ Department of Ophthalmology, Traditional Chinese Medicine Hospital of Changping District, Beijing, China

^6^ Department of Emergency, China-Japan Friendship Hospital, Beijing, China

**^#^** These authors contributed equally to this work

*** Correspondence:** Dr. Cheng Xiao: [xc2002812@126.com](mailto:xc2002812@126.com)

**Supplement table 1** Tregs in Rheumatoid Arthritis

| **Case load** | **Treg phenotype** | **Level of peripheral Tregs** | **Suppressive capacity of Tregs** | **References** |
| --- | --- | --- | --- | --- |
| 15^1^ | CD4^+^CD25^+^Foxp3^+^ | Decreased | Not tested | (Wang et al., 2012) |
| 22^2^ |  | Almost invariant | Not tested |  |
| 36^1^ | CD4^+^CD25^+^Foxp3^+^ | Decreased | Not tested | (Niu et al., 2012) |
| 30^2^ |  | Decreased | Not tested |  |
| 15 | CD4^+^CD25^+^ | Almost invariant | Decreased | (Yamagiwa et al., 2012) |
| 40 | CD4^+^CD25^+^Foxp3^+^ | Decreased | Decreased | (Chen et al., 2012) |
| 42 | CD4^+^CD25^high^Foxp3^+^ | Decreased | Not tested | (Kim et al., 2012) |
|  | CD4^+^CD25^+++^CD45RA^-^ | Decreased | Not tested |  |
|  | CD4^+^CD25^++^CD45RA^+^ | Almost invariant | Not tested |  |
| 15 | CD4^+^CD25^+^Foxp3^+^ | Decreased | Not tested | (Samson et al., 2012) |
| 50 | CD4^+^CD25^+^Foxp3^+^ | Almost invariant | Decreased | (Mcgovern et al., 2012) |
| 32 | CD4^+^CD45RA^+^Foxp3^low^ | Almost invariant | Not tested | (Matsuki et al., 2013) |
|  | CD4^+^CD45RA^-^Foxp3^high^ | Decreased | Not tested |  |
| 8 | CD4^+^ Foxp3^+^ | Decreased | Not tested | (Pesce et al., 2013) |
| 29 | CD4^+^CD25^+^CD127^low^CD45RO^+^ | Almost invariant | Increased | (Walter et al., 2013) |
| 20 | CD4^+^CD25^+^Foxp3^+^ CD127^low/-^ | Almost invariant | Decreased | (Nie et al., 2013) |
| 20 | CD4^+^CD25^high^Foxp3^+^ | Almost invariant | Not tested | (Lin et al., 2014) |
| 35 | CD4^+^CD25^+^Foxp3^+^ | Decreased | Not tested | (Gao et al., 2014) |
| 45 | CD4^+^CD25^+^Foxp3^+^ | Decreased | Not tested | (Li et al., 2014) |
| 29 | CD25^+^Foxp3^+^ CD45RA^+^ | Almost invariant | Not tested | (van der Geest KS et al., 2015) |
|  | CD25^+^Foxp3^+^ CD45RA^-^ | Decreased | Not tested |  |
| 17^5^ | CD4^+^CD25^+^Foxp3^+^ | Decreased | Not tested | (Kosmaczewska et al., 2015) |
|  | CD4^+^FoxP3^+^CTLA-4^+^ | Decreased | Not tested |  |
| 19^6^ | CD4^+^CD25^+^Foxp3^+^ | Almost invariant | Not tested |  |
|  | CD4^+^FoxP3^+^CTLA-4^+^ | Almost invariant | Not tested |  |
| 60 | CD4^+^CD25^+^Foxp3^+^ | Decreased | Not tested | (Yao et al., 2015) |
| 25 | CD4^+^CD25^+^Foxp3^+^ | Decreased | Not tested | (Zare et al., 2015) |
| 17 | CD4^+^CD25^+^Foxp3^+^ | Decreased | Decreased | (Su et al., 2016) |
| 21 | CD4^+^ Foxp3^+^ | Almost invariant | Not tested | (Ji et al., 2016) |
| 42 | CD4^+^CD25^+^CD127^low^CCR4^+^CD45RO^+^ | Almost invariant | Not tested | (Walter et al., 2016) |
| 26 | CD4^+^CD25^+^CD127^low^CXCR5^+^ | Almost invariant | Not tested | (Barbera et al., 2016) |
| 36 | CD4^+^CD25^+^Foxp3^+^ | Almost invariant | Almost invariant | (Kravchenko et al., 2016) |
| 19^1^ | CD4^+^CD25^+^Foxp3^+^ | Decreased | Decreased | (Wu et al., 2016) |
| 21^2^ | CD4^+^CD25^+^Foxp3^+^ | Almost invariant | Almost invariant |  |
| 40 | CD4^+^CD25^+^CD127^low^ | Decreased | Not tested | (Khattab et al., 2016) |
| 108 | CD3^+^CD4^+^CD25^+^CD127^low^CCR4^+^ | Almost invariant | Not tested | (Nakayamada et al., 2017) |
|  | CD3^+^CD4^+^CD25^+^CD127^low^CCR4^+^CD45RO^+^HLA-DR^+^ | Almost invariant | Not tested |  |
|  | CD3^+^CD4^+^CD25^+^CD127^low^CCR4^+^CD45RO^-^HLA-DR^-^ | Increased | Not tested |  |
|  | CD3^+^CD4^+^CD25^+^CD127^low^CCR4^+^CD45RO^+^HLA-DR^-^ | Almost invariant | Not tested |  |
| 18 | CD4^+^CD25^+^Foxp3^+^ | Decreased | Decreased | (Sun et al., 2017) |
| 85 | CD4^+^CD25^+^ | Decreased | Not tested | (Nakachi et al., 2017) |
|  | CD4^+^LAG3^+^ | Decreased | Not tested |  |
| 19 | CD4^+^CD25^+^ | Decreased | Not tested | (Dulic et al., 2017) |
| 84 | CD4^+^CD28^-^Foxp3^+^ | Increased | Decreased | (Fessler et al., 2017) |
| 39^3^ | CD45RA^-^FoxP3^+^ | Increased | Not tested | (Liu et al., 2018) |
|  | CD4^+^CXCR5^+^Foxp3^+^ | Increased | Not tested |  |
| 10^4^ | CD4^+^CXCR5^+^CD25^+^CD127^low^  CD25highCD127low | Not tested | Increased |  |
| 38 | CD4^+^CD25^+^Foxp3^+^ | Decreased | Almost invariant | (Vitales-Noyola et al., 2018) |
|  | CD4^+^CD25^var^CD69^+^LAP^+^IL-10^+^Foxp3^−^ | Increased | Decreased |  |
|  | CD4^+^CD69^+^NKG2D^+^ LAP^+^IL-10^+^Foxp3^−^ | Almost invariant | Decreased |  |
| 70 | CD4^+^CD25^+^ Foxp3^+^ CD39^+^ | Increased | Not tested | (Gupta et al., 2018) |
| 44 | CD4^+^CXCR5^+^Foxp3^+^ | Decreased | Not tested | (Niu et al., 2018) |
| 204 | CD4^+^CD25^+^Foxp3^+^ | Decreased | Decreased | (Hashemi et al., 2018) |
| 25 | CD4^+^CD25^+^Foxp3^+^ | Decreased | Not tested | (Wang et al., 2018) |
| 24 | CD4^+^CXCR5^+^CD127^low^ | Increased | Not tested | (Wang et al., 2019) |
| 8 | CD4^+^CD25^+^ | Almost invariant | Not tested | (Kailashiya et al., 2019) |
|  | CD4^+^CD25^+^CD127^low^ | Increased | Not tested |  |
| 74 | CD4^+^CD25^+^CD127^low/-^Foxp3^+^ | Almost invariant | Not tested | (Yang et al., 2019) |
|  | CD4^+^CD25^+^CD127^low/-^Foxp3^+^Helios^+^ | Decreased | Not tested |  |
|  | CD4^+^CD25^+^CD127^low/-^Foxp3^+^CD226^+^ | Increased | Not tested |  |
|  | CD4^+^CD25^+^CD127^low/-^Foxp3^+^TIGIT^+^ | Increased | Not tested |  |
| 65 | CD4^+^CD25^high^CD127^low^ | Decreased | Decreased | (Huang et al., 2020) |
| 45 | CD4^+^CD25^high^CD127^-^ | Decreased | Not tested | (Paradowska-Gorycka et al., 2020) |
| 55 | CD4^+^CD25^+^Foxp3^+^ | Decreased | Not tested | (Niu et al., 2020) |
| 42 | CD4^+^CD25^+^ | Decreased | Not tested | (Farid et al., 2020) |
| 45 | CD4^+^Foxp3^+^ | Decreased | Decreased | (Avdeeva et al., 2020) |
| 14^2^ | CD4^+^CD25^+^CD127^low/-^ | Decreased | Almost invariant | (Kanjana et al., 2020) |
| 13^1^ | CD4^+^CD25^+^CD127^low/-^ | Decreased | Decreased |  |
| 19 | CD4^+^CD25^+^CD127^low/-^ | Decreased | Decreased | (Li et al., 2020) |
| 20 | CD4^+^CD25^+^ | Decreased | Not tested | (Wan et al., 2020) |
| 21 | CD4^+^CD25^high^Foxp3^+^ | Decreased | Almost invariant | (Shevyrev et al., 2021) |
|  | CD4^+^CD25^+^Foxp3^+^ | Almost invariant | Almost invariant |  |
| 888 | CD4^+^CD25^+^Foxp3^+^ | Decreased | Not tested | (Zhang et al., 2021) |
| 13 | CD4^+^CD25^+^ | Almost invariant | Decreased | (Go et al., 2021) |
|  | CD4^+^ Foxp3^+^ | Almost invariant | Decreased |  |
|  | CD4^+^CD25^+^Foxp3^+^ | Almost invariant | Decreased |  |
|  | CD4^+^CD25^+^CD127^low/-^ | Almost invariant | Decreased |  |
|  | CD4^+^CD25^+^CD127^low/-^Foxp3^+^ | Almost invariant | Decreased |  |
|  | CD4+CD25^int^CD45RA^+^ | Almost invariant | Decreased |  |
|  | CD4^+^CD25^high^CD45RA^−^ | Decreased | Decreased |  |
|  | CD4^+^CD25^int^CD45RA^−^ | Almost invariant | Almost invariant |  |

^1^ active RA

^2^ stable remission

^3^ 39 patients with active RA, 39 patients with RA in stable remission

^4^ 10 patients with active RA, 10 patients with RA in stable remission

^5^ mean (range) disease duration of 123.7 (13 to 300) months

^6^ mean (range) RA duration of 15.2 (2 to 79) months

**References**

Avdeeva, A., Rubtsov, Y., Dyikanov, D., Popkova, T., and Nasonov, E. (2020). Regulatory T cells in patients with early untreated rheumatoid arthritis: Phenotypic changes in the course of methotrexate treatment. Biochimie*.* 174, 9-17. doi: 10.1016/j.biochi.2020.03.014

Barbera, A., Lorenzo, N., van Kooten, P., van Roon, J., de Jager, W., Prada, D., Gomez, J., Padron, G., van Eden, W., Broere, F., and Del, C. D. M. (2016). APL1, an altered peptide ligand derived from human heat-shock protein 60, increases the frequency of Tregs and its suppressive capacity against antigen responding effector CD4 + T cells from rheumatoid arthritis patients. Cell Stress Chaperones*.* 21(4), 735-44. doi: 10.1007/s12192-016-0698-0

Chen, R., Tao, Y., Qiu, K., Huang, W., Huang, C., and Li, J. (2012). [Association of circulating Treg cells with disease activity in patients with rheumatoid arthritis]. Nan Fang Yi Ke Da Xue Xue Bao*.* 32(6), 886-9

Dulic, S., Vasarhelyi, Z., Sava, F., Berta, L., Szalay, B., Toldi, G., Kovacs, L., and Balog, A. (2017). T-Cell Subsets in Rheumatoid Arthritis Patients on Long-Term Anti-TNF or IL-6 Receptor Blocker Therapy. Mediators Inflamm*.* 2017, 6894374. doi: 10.1155/2017/6894374

Farid, E., Mumtaz, M., Hajji, F., Ebrahim, R. A., Abdulla, H., and Tabbara, K. (2020). T Regulatory Cells in Rheumatoid Arthritis with Reference to Anti-Citrullinated Peptide Antibody and TNF-alpha Inhibitor Therapy. Egypt J Immunol*.* 27(1), 55-63

Fessler, J., Raicht, A., Husic, R., Ficjan, A., Schwarz, C., Duftner, C., Schwinger, W., Graninger, W. B., Stradner, M. H., and Dejaco, C. (2017). Novel Senescent Regulatory T-Cell Subset with Impaired Suppressive Function in Rheumatoid Arthritis. Front Immunol*.* 8, 300. doi: 10.3389/fimmu.2017.00300

Gao, S., Hao, B., Yang, X. F., and Chen, W. Q. (2014). Decreased CD200R expression on monocyte-derived macrophages correlates with Th17/Treg imbalance and disease activity in rheumatoid arthritis patients. Inflamm. Res. 63(6), 441-450

Go, E., Yoo, S. J., Choi, S., Sun, P., Jung, M. K., Kwon, S., Heo, B. Y., Kim, Y., Kang, J. G., Kim, J., Shin, E. C., Kang, S. W., and Kwon, J. (2021). Peripheral Blood from Rheumatoid Arthritis Patients Shows Decreased Treg CD25 Expression and Reduced Frequency of Effector Treg Subpopulation. Cells*.* 10(4). doi: 10.3390/cells10040801

Gupta, V., Katiyar, S., Singh, A., Misra, R., and Aggarwal, A. (2018). CD39 positive regulatory T cell frequency as a biomarker of treatment response to methotrexate in rheumatoid arthritis. Int. J. Rheum. Dis. 21(8), 1548-1556. doi: 10.1111/1756-185X.13333

Hashemi, V., Farrokhi, A. S., Tanomand, A., Babaloo, Z., Hojjat-Farsangi, M., Anvari, E., Tahoori, M. T., Ezzeddini, R., Hosseini, A., Gharibi, T., Ghalamfarsa, G., and Jadidi-Niaragh, F. (2018). Polymorphism of Foxp3 gene affects the frequency of regulatory T cells and disease activity in patients with rheumatoid arthritis in Iranian population. Immunol. Lett. 204, 16-22. doi: 10.1016/j.imlet.2018.10.001

Huang, Y., Wang, H., Ba, X., Chen, Z., Wang, Y., Qin, K., Huang, Y., Shen, P., and Tu, S. (2020). Decipher manifestations and Treg /Th17 imbalance in multi-staging rheumatoid arthritis and correlation with TSDR/RORC methylation. Mol. Immunol. 127, 1-11. doi: 10.1016/j.molimm.2020.08.002

Ji, L., Geng, Y., Zhou, W., and Zhang, Z. (2016). A study on relationship among apoptosis rates, number of peripheral T cell subtypes and disease activity in rheumatoid arthritis. Int. J. Rheum. Dis. 19(2), 167-171

Kailashiya, V., Singh, U., Rana, R., Singh, N. K., Dash, D., and Kailashiya, J. (2019). Regulatory T Cells and Their Association with Serum Markers and Symptoms in Systemic Lupus Erythematosus and Rheumatoid Arthritis. Immunol. Invest. 48(1), 64-78. doi: 10.1080/08820139.2018.1527852

Kanjana, K., Chevaisrakul, P., Matangkasombut, P., Paisooksantivatana, K., and Lumjiaktase, P. (2020). Inhibitory activity of FOXP3+ regulatory T cells reveals high specificity for displaying immune tolerance in remission state rheumatoid arthritis. Sci Rep*.* 10(1), 19789. doi: 10.1038/s41598-020-76168-1

Khattab, S. S., El-Saied, A. M., Mohammed, R. A., and Mohamed, E. E. (2016). CD4+ CD25+ CD127low Regulatory T Cells as Indicator of Rheumatoid Arthritis Disease Activity. Egypt J Immunol*.* 23(2), 87-95

Kim, J. R., Chae, J. N., Kim, S. H., and Ha, J. S. (2012). Subpopulations of regulatory T cells in rheumatoid arthritis, systemic lupus erythematosus, and Behcet's disease. J. Korean Med. Sci. 27(9), 1009-13. doi: 10.3346/jkms.2012.27.9.1009

Kosmaczewska, A., Ciszak, L., Swierkot, J., Szteblich, A., Kosciow, K., and Frydecka, I. (2015). Exogenous IL-2 Controls the Balance in Th1, Th17, and Treg Cell Distribution in Patients with Progressive Rheumatoid Arthritis Treated with TNF-Alpha Inhibitors. Inflammation*.* 38(2), 765-774

Kravchenko, P. N., Zhulai, G. A., Churov, A. V., Oleinik, E. K., Oleinik, V. M., Barysheva, O. Y., Vezikova, N. N., and Marusenko, I. M. (2016). [Subpopulations of Regulatory T-lymphocytes in the Peripheral Blood of Patients with Rheumatoid Arthritis]. Vestn Ross Akad Med Nauk(2), 148-53. doi: 10.15690/vramn656

Li, M., Ma, K., Feng, Z., Wang, J., Zhou, X., and Zhou, L. (2020). Differential long non-coding RNA expression profiles in the peripheral blood and CD4(+) T cells of patients with active rheumatoid arthritis. Exp. Ther. Med. 20(1), 461-471. doi: 10.3892/etm.2020.8681

Li, N., Ma, T., Han, J., Zhou, J., Wang, J., Zhang, J., and Zheng, S. (2014). Increased apoptosis induction in CD4+ CD25+ Foxp3+ T cells contributes to enhanced disease activity in patients with rheumatoid arthritis through IL-10 regulation. Eur Rev Med Pharmacol Sci*.* 18(1), 78-85

Lin, H., Zhang, G. D., Tang, H. H., Wang, Y., Liu, Y., and Zhao, Y. (2014). [The change of CD4+ CD25+ regulatory T cells in patients with rheumatoid arthritis]. Sichuan Da Xue Xue Bao Yi Xue Ban*.* 45(4), 618-22

Liu, C., Wang, D., Lu, S., Xu, Q., Zhao, L., Zhao, J., Song, Y., and Wang, H. (2018). Increased Circulating Follicular Treg Cells Are Associated With Lower Levels of Autoantibodies in Patients With Rheumatoid Arthritis in Stable Remission. Arthritis Rheumatol*.* 70(5), 711-721. doi: 10.1002/art.40430

Matsuki, F., Saegusa, J., Miyamoto, Y., Misaki, K., Kumagai, S., and Morinobu, A. (2013). CD45RA-Foxp3(high) activated/effector regulatory T cells in the CCR7 + CD45RA-CD27 + CD28+central memory subset are decreased in peripheral blood from patients with rheumatoid arthritis. Biochem Biophys Res Commun*.* 438(4), 778-83. doi: 10.1016/j.bbrc.2013.05.120

Mcgovern, J. L., Nguyen, D. X., Notley, C. A., Mauri, C., Isenberg, D. A., and Ehrenstein, M. R. (2012). Th17 cells are restrained by Treg cells via the inhibition of interleukin-6 in patients with rheumatoid arthritis responding to anti-tumor necrosis factor antibody therapy. Arthritis Rheum*.* 64(10), 3129-38. doi: 10.1002/art.34565

Nakachi, S., Sumitomo, S., Tsuchida, Y., Tsuchiya, H., Kono, M., Kato, R., Sakurai, K., Hanata, N., Nagafuchi, Y., Tateishi, S., Kanda, H., Okamura, T., Yamamoto, K., and Fujio, K. (2017). Interleukin-10-producing LAG3(+) regulatory T cells are associated with disease activity and abatacept treatment in rheumatoid arthritis. Arthritis Res. Ther. 19(1), 97. doi: 10.1186/s13075-017-1309-x

Nakayamada, S., Kubo, S., Yoshikawa, M., Miyazaki, Y., Yunoue, N., Iwata, S., Miyagawa, I., Hirata, S., Nakano, K., and Saito, K. (2017). Differential effects of biological DMARDs on peripheral immune cell phenotypes in patients with rheumatoid arthritis. Rheumatology

Nie, H., Zheng, Y., Li, R., Guo, T. B., He, D., Fang, L., Liu, X., Xiao, L., Chen, X., Wan, B., Chin, Y. E., and Zhang, J. Z. (2013). Phosphorylation of FOXP3 controls regulatory T cell function and is inhibited by TNF-alpha in rheumatoid arthritis. Nat. Med. 19(3), 322-8. doi: 10.1038/nm.3085

Niu, H. Q., Li, Z. H., Zhao, W. P., Zhao, X. C., Zhang, C., Luo, J., Lu, X. C., Gao, C., Wang, C. H., and Li, X. F. (2020). Sirolimus selectively increases circulating Treg cell numbers and restores the Th17/Treg balance in rheumatoid arthritis patients with low disease activity or in DAS28 remission who previously received conventional disease-modifying anti-rheumatic drugs. Clin. Exp. Rheumatol. 38(1), 58-66

Niu, Q., Cai, B., Huang, Z. C., Shi, Y. Y., and Wang, L. L. (2012). Disturbed Th17/Treg balance in patients with rheumatoid arthritis. Rheumatol. Int. 32(9), 2731-6. doi: 10.1007/s00296-011-1984-x

Niu, Q., Huang, Z. C., Wu, X. J., Jin, Y. X., An, Y. F., Li, Y. M., Xu, H., Yang, B., and Wang, L. L. (2018). Enhanced IL-6/phosphorylated STAT3 signaling is related to the imbalance of circulating T follicular helper/T follicular regulatory cells in patients with rheumatoid arthritis. Arthritis Res. Ther. 20(1), 200. doi: 10.1186/s13075-018-1690-0

Paradowska-Gorycka, A., Wajda, A., Romanowska-Prchnicka, K., Walczuk, E., Kuca-Warnawin, E., Kmiolek, T., Stypinska, B., Rzeszotarska, E., Majewski, D., Jagodzinski, P. P., and Pawlik, A. (2020). Th17/Treg-Related Transcriptional Factor Expression and Cytokine Profile in Patients With Rheumatoid Arthritis. Front Immunol*.* 11, 572858. doi: 10.3389/fimmu.2020.572858

Pesce, B., Soto, L., Sabugo, F., Wurmann, P., Cuchacovich, M., Lopez, M. N., Sotelo, P. H., Molina, M. C., Aguillon, J. C., and Catalan, D. (2013). Effect of interleukin-6 receptor blockade on the balance between regulatory T cells and T helper type 17 cells in rheumatoid arthritis patients. Clin. Exp. Immunol. 171(3), 237-42. doi: 10.1111/cei.12017

Samson, M., Audia, S., Janikashvili, N., Ciudad, M., Trad, M., Fraszczak, J., Ornetti, P., Maillefert, J. F., Miossec, P., and Bonnotte, B. (2012). Brief report: inhibition of interleukin-6 function corrects Th17/Treg cell imbalance in patients with rheumatoid arthritis. Arthritis Rheum*.* 64(8), 2499-503. doi: 10.1002/art.34477

Shevyrev, D., Tereshchenko, V., Kozlov, V., Sizikov, A., Chumasova, O., and Koksharova, V. (2021). T-regulatory cells from patients with rheumatoid arthritis retain suppressor functions in vitro. Exp. Ther. Med. 21(3), 209. doi: 10.3892/etm.2021.9641

Su, D., Shen, M., Gu, B., Wang, X., Wang, D., Li, X., and Sun, L. (2016). (99) Tc-methylene diphosphonate improves rheumatoid arthritis disease activity by increasing the frequency of peripheral gammadelta T cells and CD4(+) CD25(+) Foxp3(+) Tregs. Int. J. Rheum. Dis. 19(6), 586-93. doi: 10.1111/1756-185X.12292

Sun, H., Gao, W., Pan, W., Zhang, Q., Wang, G., Feng, D., Geng, X., Yan, X., and Li, S. (2017). Tim3(+) Foxp3 (+) Treg Cells Are Potent Inhibitors of Effector T Cells and Are Suppressed in Rheumatoid Arthritis. Inflammation*.* 40(4), 1342-1350. doi: 10.1007/s10753-017-0577-6

van der Geest KS, K, S., Ja, P., Wh, A., Hw, K., Bj, K., van den Berg A, Am, B., Eb, L., and E, B. (2015). SF Treg cells transcribing high levels of Bcl-2 and microRNA-21 demonstrate limited apoptosis in RA. Rheumatology (Oxford, England)*.* 54(5), 950-8. doi: 10.1093/rheumatology/keu407

Vitales-Noyola, M., Layseca-Espinosa, E., Baranda, L., Abud-Mendoza, C., Nino-Moreno, P., Monsivais-Urenda, A., Rosenstein, Y., and Gonzalez-Amaro, R. (2018). Analysis of Sodium Chloride Intake and Treg/Th17 Lymphocytes in Healthy Individuals and Patients with Rheumatoid Arthritis or Systemic Lupus Erythematosus. J Immunol Res*.* 2018, 9627806. doi: 10.1155/2018/9627806

Walter, G. J., Evans, H. G., Menon, B., Gullick, N. J., Kirkham, B. W., Cope, A. P., Geissmann, F., and Taams, L. S. (2013). Interaction with activated monocytes enhances cytokine expression and suppressive activity of human CD4+CD45ro+CD25+CD127(low) regulatory T cells. Arthritis Rheum*.* 65(3), 627-38. doi: 10.1002/art.37832

Walter, G. J., Fleskens, V., Frederiksen, K. S., Rajasekhar, M., Menon, B., Gerwien, J. G., Evans, H. G., and Taams, L. S. (2016). Phenotypic, Functional, and Gene Expression Profiling of Peripheral CD45RA+ and CD45RO+ CD4+CD25+CD127(low) Treg Cells in Patients With Chronic Rheumatoid Arthritis. Arthritis Rheumatol*.* 68(1), 103-16. doi: 10.1002/art.39408

Wan, L., Liu, J., Huang, C., Chen, X., Zhao, L., Fan, H., Ge, Y., Liu, T., and Liu, L. (2020). [Inflammation caused by different immune cell subsets is involved in bone destruction of rheumatoid arthritis]. Xi Bao Yu Fen Zi Mian Yi Xue Za Zhi*.* 36(11), 1026-1031

Wang, L., Wang, C., Jia, X., and Yu, J. (2018). Circulating Exosomal miR-17 Inhibits the Induction of Regulatory T Cells via Suppressing TGFBR II Expression in Rheumatoid Arthritis. Cell. Physiol. Biochem. 50(5), 1754-1763. doi: 10.1159/000494793

Wang, W., Shao, S., Jiao, Z., Guo, M., Xu, H., and Wang, S. (2012). The Th17/Treg imbalance and cytokine environment in peripheral blood of patients with rheumatoid arthritis. Rheumatol. Int. 32(4), 887-93. doi: 10.1007/s00296-010-1710-0

Wang, X., Yang, C., Xu, F., Qi, L., Wang, J., and Yang, P. (2019). Imbalance of circulating Tfr/Tfh ratio in patients with rheumatoid arthritis. Clin. Exp. Med. 19(1), 55-64. doi: 10.1007/s10238-018-0530-5

Wu, Y. H., Liu, W., Xue, B., Zhang, L., Liu, X. Y., Liu, B., Wang, Y., Cai, Y., and Duan, R. (2016). Upregulated Expression of microRNA-16 Correlates with Th17/Treg Cell Imbalance in Patients with Rheumatoid Arthritis. DNA Cell Biol. 35(12), 853-860. doi: 10.1089/dna.2016.3349

Yamagiwa, T., Fukunishi, S., Tachibana, T., Okamura, H., Yoshiya, S., and Kashiwamura, S. (2012). Abrogation of Treg function deteriorates rheumatoid arthritis. Mod. Rheumatol. 22(1), 80-8. doi: 10.1007/s10165-011-0476-x

Yang, M., Liu, Y., Mo, B., Xue, Y., Ye, C., Jiang, Y., Bi, X., Liu, M., Wu, Y., Wang, J., Olsen, N., Pan, Y., and Zheng, S. G. (2019). Helios but not CD226, TIGIT and Foxp3 is a Potential Marker for CD4(+) Treg Cells in Patients with Rheumatoid Arthritis. Cell. Physiol. Biochem. 52(5), 1178-1192. doi: 10.33594/000000080

Yao, X., Cao, Y., Ma, W., Hou, L., Zhong, Q., Huang, Y., Tang, F., Xu, H., and Liang, J. (2015). [Elevated levels of Th17/regulatory T cells in peripheral blood are associated with disease activity of patients with rheumatoid arthritis]. Xi Bao Yu Fen Zi Mian Yi Xue Za Zhi*.* 31(1), 81-4

Zare, H. R., Habibagahi, M., Vahdati, A., and Habibagahi, Z. (2015). Patients with Active Rheumatoid Arthritis Have Lower Frequency of nTregs in Peripheral Blood. Iran. J. Immunol. 12(3), 166-75. doi: IJIv12i3A2

Zhang, S. X., Wang, J., Wang, C. H., Jia, R. H., Yan, M., Hu, F. Y., Liu, G. Y., Liu, X. Y., Luo, J., Gao, C., and Li, X. F. (2021). Low-dose IL-2 therapy limits the reduction in absolute numbers of circulating regulatory T cells in rheumatoid arthritis. Ther Adv Musculoskelet Dis*.* 13, 1759720X211011370. doi: 10.1177/1759720X211011370
